# Erratum to “Alanine aminotransferase contributes to hypoxia sensitivity and dormancy in barley seeds”

**DOI:** 10.1002/tpg2.70081

**Published:** 2025-07-21

Farquharson, L. G. H., Samanfar, B., Khanal, R., & Brauer, E. K. (2025). Alanine aminotransferase contributes to hypoxia sensitivity and dormancy in barley seeds. *The Plant Genome, 18*, e70063. https://doi.org/10.1002/tpg2.70063

In the second sentence of the Abstract, the text “In barley (*Hordeum vulgare* L.), the SD1 and SD2 (where SD is standard deviation) loci regulate dormancy and pre‐harvest sprouting (PHS), though their role in physiological development remains unclear.” was incorrect. This should have read: “In barley (*Hordeum vulgare* L.), the SD1 and SD2 (where SD is seed dormancy) loci regulate dormancy and pre‐harvest sprouting (PHS), though their role in physiological development remains unclear.”

In the Abbreviations list, the text “SD, standard deviation” was incorrect. The text should have read: “SD1, seed dormancy 1; SD2, seed dormancy 2.”

In the third paragraph under the Introduction section, the text “Previous efforts to establish genetic regulation of dormancy in barley have identified the SD1 and SD2 (where SD is standard deviation) loci in multiple barley populations…” was incorrect. This should have read: “Previous efforts to establish genetic regulation of dormancy in barley have identified the SD1 and SD2 (where SD is seed dormancy) loci in multiple barley populations…”.

In the first sentence under Core Ideas, the text “A QTLs proximal to the SD1 (where SD is standard deviation) locus regulates dormancy in a Canadian barley population.” was incorrect. This should have read: “A QTL proximal to the SD1 (where SD is seed dormancy) locus regulates dormancy in a Canadian barley population.”

In the first sentence of the caption for Table 1, the text: “SD1 and SD2 (where SD is standard deviation) allele characterization of barley genotypes.” was incorrect. The caption should have read: “SD1 and SD2 (where SD is seed dormancy) allele characterization of barley genotypes.”

We apologize for these errors.

